# Identification of Galectin-3 as Potential Biomarkers for Renal Fibrosis by RNA-Sequencing and Clinicopathologic Findings of Kidney Biopsy

**DOI:** 10.3389/fmed.2021.748225

**Published:** 2021-11-12

**Authors:** Shuo-Ming Ou, Ming-Tsun Tsai, Huan-Yuan Chen, Fu-An Li, Wei-Cheng Tseng, Kuo-Hua Lee, Fu-Pang Chang, Yao-Ping Lin, Ruey-Bing Yang, Der-Cherng Tarng

**Affiliations:** ^1^Division of Nephrology, Department of Medicine, Taipei Veterans General Hospital, Taipei, Taiwan; ^2^School of Medicine, National Yang-Ming University, Taipei, Taiwan; ^3^School of Medicine, National Yang Ming Chiao Tung University, Taipei, Taiwan; ^4^Institute of Clinical Medicine, National Yang-Ming University, Taipei, Taiwan; ^5^Institute of Clinical Medicine, National Yang Ming Chiao Tung University, Taipei, Taiwan; ^6^Center for Intelligent Drug Systems and Smart Bio-devices (IDS2B), National Yang Ming Chiao Tung University, Hsinchu, Taiwan; ^7^Institute of Biomedical Sciences, Academia Sinica, Taipei, Taiwan; ^8^Department of Pathology and Laboratory Medicine, Taipei Veterans General Hospital, Taipei, Taiwan; ^9^Inflammation and Immunity Research Center, National Yang-Ming University, Taipei, Taiwan; ^10^Department and Institute of Physiology, National Yang-Ming University, Taipei, Taiwan; ^11^Department and Institute of Physiology, National Yang Ming Chiao Tung University, Taipei, Taiwan

**Keywords:** galectin-3, chronic kidney disease, tubular atrophy, interstitial fibrosis, kidney biopsy, RNA-sequencing analysis

## Abstract

**Background:** Galectin-3 (Gal-3) is a multifunctional glycan-binding protein shown to be linked to chronic inflammation and fibrogenesis. Plasma Gal-3 is associated with proteinuria and renal dysfunction, but its role has never been confirmed with kidney biopsy results. In our study, we aimed to explore the expression of Gal-3 in biopsy-proven patients, and we tested the hypothesis that chronic kidney disease (CKD) leads to upregulation of plasma Gal-3 expression in corresponding biopsy findings and RNA sequencing analysis.

**Method:** In 249 patients (male/female: 155/94, age: 57.2 ± 16.3 years) who underwent kidney biopsy, plasma levels of Gal-3 were measured to estimate the association of renal fibrosis. Relationships between plasma Gal-3 levels, estimated glomerular filtration rate (eGFR) and renal histology findings were also assessed. We further examined the gene expression of Gal-3 in RNA-sequencing analysis in biopsy-proven patients.

**Results:** Compared to patients without CKD, CKD patients had higher levels of plasma Gal-3 (1,016.3 ± 628.1 pg/mL vs. 811.6 ± 369.6 pg/ml; *P* = 0.010). Plasma Gal-3 was inversely correlated with eGFR (*P* = 0.005) but not with proteinuria. Higher Gal-3 levels were associated with interstitial fibrosis, tubular atrophy and vascular intimal fibrosis. RNA-sequencing analysis showed the upregulation of Gal-3 in fibrotic kidney biopsy samples, and the differentially expressed genes were mainly enhanced in immune cell activation and the regulation of cell-cell adhesion.

**Conclusions:** Plasma Gal-3 levels are inverse correlated with eGFR but positively correlated with renal fibrosis, which may be involved in the immune response and associated pathways. These findings support the role of Gal-3 as a predictive marker of renal fibrosis.

## Introduction

Chronic kidney disease (CKD) remains a major public health problem and is one of the leading causes of mortality and morbidity worldwide (1–3). CKD patients are generally asymptomatic, so it is difficult to recognize the early clinical signs or symptoms of CKD (4). Current biomarkers, such as serum blood urea nitrogen, serum creatinine and proteinuria, lack sensitivity for the early detection of the development of CKD (5, 6). Interstitial fibrosis is characterized by the accumulation of collagen-rich extracellular matrix in the interstitium (7, 8). Tubular atrophy is defined as the presence of tubular epithelial thinning with pyknotic nuclei or dilated and thin tubules (9). Interstitial fibrosis and tubular atrophy are considered as the common pathway for CKD progression (9–11). If we can identify these patients in the early disease stage, their renal function decline may be slowed or even reversed. Therefore, it is important to find useful predictive biomarkers to identify individuals at risk for renal fibrosis at the earliest possible stage (12).

Human galectin-3 (Gal-3) is a β-galactoside-binding lectin protein encoded by a single gene (LGALS3) located on chromosome 14 (13). Gal-3 plays a role in signaling in angiogenesis, oncogenic transformation, inflammatory responses and fibrogenic processes (14, 15). Upregulation of Gal-3 expression has been considered a key regulator of inflammation and fibrosis and may contribute to multiple organ fibrosis (16–18). In an *in vitro* model, Gal-3^−/−^ fibroblasts showed reduced collagen production in response to transforming growth factor-beta 1 (TGF-β1). Moreover, in an *in vivo* model, Gal-3 knockout mice exhibited a decrease in collagen accumulation and resistance to the development of fibrosis (19, 20). Because there is increasing evidence that Gal-3 is associated with the development of organ fibrosis (21), Gal-3 may serve as a potential biomarker for renal fibrosis through its possible pathogenic mechanisms of action.

In the general population, data from the Framingham Offspring Study, which involved 2,450 subjects, showed that higher plasma levels of Gal-3 were associated with a higher risk of CKD but not with the risk of albuminuria (21). Other studies found that higher plasma Gal-3 levels were associated with poorer survival, renal insufficiency and late allograft failure (22, 23). However, another study consisting of 7,968 subjects from the Prevention of REnal and Vascular ENd-stage Disease (PREVEND) cohort found that the correlation between Gal-3 and renal function was weak (24). Because some inconsistent results exist, this topic deserves further investigation of the links between plasma Gal-3 levels, Gal-3 gene expression and detailed renal pathologic findings.

To address this problem, we performed a prospective study on patients who underwent kidney biopsy to examine the association between their plasma Gal-3 levels and renal histopathology findings. In addition, we examined the gene expression of Gal-3 using RNA-sequencing analysis in kidney specimens. The present study aimed to (1) characterize the associations between plasma Gal-3 and clinical variables and laboratory results, (2) investigate whether plasma Gal-3 was associated with the pathological findings of CKD, such as renal fibrosis and/or tubular atrophy, and (3) identify the gene expression of Gal-3 using RNA-sequencing analysis in kidney biopsy specimens. We also attempted to perform Gene Ontology (GO) and Kyoto Encyclopedia of Genes and Genomes (KEGG) pathway enrichment analyses to explore the possible pathogenesis and potential pathways of Gal-3 in the development of renal fibrosis.

## Methods

### Study Design and Population

All patients who underwent renal biopsies at the Taipei Veteran General Hospitals between 2018 and 2021 were included in this cohort study. Patients who were younger than 20 years of age, who were unable or unwilling to provide signed informed consent or who were missing plasma Gal-3 measurements were excluded from our study. Finally, 249 patients who underwent renal biopsies and signed the informed consent form were enrolled. The study protocol was approved by the Institutional Review Board of the Taipei Veteran General Hospitals (2018-06-008B).

### Clinical Variables and Data Collection

Information for all enrolled patients, including demographic data, underlying comorbidities and laboratory results, was collected. The demographic data included age, sex, body mass index [BMI, calculated using the following formula: weight (kg)/height^2^ (m^2^)], systolic blood pressure, and diastolic blood pressure. The underlying comorbidities included diabetes mellitus, coronary artery disease, congestive heart failure, hypertension, dyslipidemia and malignancy. The laboratory parameters assessed were low-density lipoprotein cholesterol, uric acid, albumin, and eGFR. Urinary samples were collected from the patients, and the urine albumin-to-creatinine ratio (UACR) and urine protein-to-creatinine ratio (UPCR) were calculated.

### Plasma Gal-3 Measurement and Outcome Definitions

Blood samples were collected at the time of renal biopsy, immediately centrifuged and stored frozen at −80°C until assayed. The plasma Gal-3 concentration was measured as picograms per milliliter (pg/mL) using an inhouse multiplex bead-based immunoassay (25, 26). The estimated GFR (eGFR) was calculated by using the CKD Epidemiology Collaboration equation (27, 28).

### Histopathologic Analysis of Kidney Biopsies

Kidney biopsies were reviewed by one pathologist using light microscopy (LM), immunohistochemistry and electron microscopy. Formalin-fixed and paraffin-embedded kidney biopsy specimens were used for LM. The kidney specimens sent for LM were stained with hematoxylin and eosin (H&E), periodic acid–Schiff (PAS) and Masson's trichrome stains. Immunofluorescence staining was performed on frozen kidney specimen sections, which were then examined for immunoglobulin or complement deposits inside the tissues. Electron microscopy examinations of the kidney specimens were performed to observe the ultrastructure of the glomerulus and tubulointerstitium. Pathological findings, including glomerular descriptions (e.g., global sclerosis, segmental sclerosis, double contour of glomerular basement membrane [GBM], GBM rigid, GBM thickening, GBM collapse, GBM attenuation, glomerular necrosis, glomerular inflammatory change, glomerular ischemic change, endocapillary hypertrophy, extracapillary hypertrophy, mesangial hypertrophy, mesangial matrix expansion, mesangial matrix mesangiolysis), tubulointerstitial descriptions (e.g., interstitial inflammation, tubulitis, interstitial edema, interstitial fibrosis, tubular atrophy, acute tubular necrosis, casts and calcinosis) and vasculature descriptions (e.g., hyaline arteriosclerosis, vascular intimal fibrosis, vascular medial proliferation, and vascular necrosis) were examined.

### RNA-Sequencing Analysis and Identification of Differentially Expressed Genes (DEGs)

After kidney biopsy specimens were obtained, kidney biopsy specimens were storage in RNAlater and then frozen in −80°C until microdissection. During microdissection, the morphology of glomeruli was identified and carefully picked up by fine forceps in microscopy. After then, the renal tubulointerstitial segments were sent for RNA-sequencing analysis. RNA was extracted using the RNeasy mini kit, and RNA sequencing was performed on the Illumina NovaSeq 6000 platform at 150 bp paired end (29). The annotated RNA counts (fastq) were calculated, and sequence quality was also surveyed with FastQC (30). After trimming adaptors and lower-quality bases, the DEGs among fibrotic groups and control groups were identified using R software with a limma package from the Bioconductor project (31). Fold-changes (FCs) in the gene expression values were calculated, and a FC > 2 and *P*-value < 0.05 were considered the cutoff values for identifying DEGs.

### GO and KEGG Enrichment Analyses

GO is a structured, controlled vocabulary to unify the representation of genes and classify gene functions. The enriched GO terms included three non-overlapping ontologies, including biological process (BP), cellular component (CC) and molecular function (MF) (32, 33). KEGG is a collection of databases for systematic analysis of gene functions and biological systems, which includes most of the known metabolic and gene regulatory pathways for data visualization (34). We conducted GO enrichment and KEGG analyses of the DEGs by using the clusterProfiler package in R software.

### Statistical Analyses

In our study, plasma Gal-3 was considered to be a continuous variable and a categorical variable according to the tertile of plasma Gal-3 levels. Correlations between Gal-3, eGFR, serum creatinine, UPCR and UACR were assessed using Pearson's correlation. We also analyzed non-linear associations between plasma Gal-3 levels and eGFR change by restricted cubic spline plots with three knots using tertile of plasma Gal-3 levels. The statistical significance was *P* < 0.05. Data were analyzed using SAS software (version 9.4; SAS Institute Inc., Cary, NC, USA) and R software (version 3.5.2 for Windows).

## Results

### Patient Characteristics

The demographic and clinical characteristics of our patients are shown in Table 1. Of the 249 study participants, we divided the study participants into three groups based on the tertile of plasma Gal-3 levels (<610.7, 610.8–1039.8, and >1039.9 pg/mL, respectively). The mean (SD) age of the patients was 57.2 (16.3) years and 62.3% were male. Among them, 64 (25.7%) patients had diabetes mellitus and 105 (42.2%) patients had hypertension. The UACR and UPCR did not differ between the groups.

**Table 1:** Clinical characteristics of our study participants stratified by tertile of plasma Gal-3 levels.

		**Tertile of plasma Gal-3 levels**			
	**All patients**	**Lowest tertile**	**Middle tertile**	**Highest tertile**	***P*-value**
	**(*n* = 249)**	**(<610.7 pg/mL)**	**(610.8–1039.8 pg/mL)**	**(>1039.9 pg/mL)**	
		**(*n* = 83)**	**(*n* = 83)**	**(*n* = 83)**	
Age, years	57.2 ± 16.3	54.3 ± 16.8	58.2 ± 17.1	59.0 ± 14.7	0.136
Male sex, *n* (%)	155 (62.3)	48 (57.8)	52 (62.7)	55 (66.3)	0.531
Body mass index	25.6 ± 4.6	25.9 ± 4.3	25.5 ± 5.0	25.3 ± 4.4	0.679
Systolic blood pressure, mmHg	136.8 ± 22.7	136.5 ± 24.2	133.2 ± 21.6	140.7 ± 22.0	0.106
Diastolic blood pressure, mmHg	77.2 ± 13.0	75.6 ± 12.5	77.4 ± 13.1	78.4 ± 13.5	0.370
Diabetes mellitus, *n* (%)	64 (25.7)	23 (27.7)	14 (16.9)	27 (32.5)	0.061
Coronary artery disease, *n* (%)	20 (8.0)	6 (7.2)	4 (4.8)	10 (12.0)	0.218
Congestive heart failure, *n* (%)	46 (18.5)	17 (20.5)	12 (14.5)	17 (20.5)	0.513
Hypertension, *n* (%)	105 (42.2)	31 (37.3)	31 (37.3)	43 (51.8)	0.093
Dyslipidemia, *n* (%)	47 (18.9)	17 (20.5)	17 (20.5)	13 (15.7)	0.657
Malignancy, *n* (%)	60 (24.1)	21 (25.3)	16 (19.3)	23 (27.7)	0.425
LDL-C, mg/dL	140.5 ± 85.6	142.6 ± 88.3	156.9 ± 98.6	120.4 ± 60.9	0.038^†^
Uric acid, mg/dL	6.7 ± 2.2	6.7 ± 2.1	6.5 ± 2.2	7.1 ± 2.3	0.289
Albumin, mg/dL	3.2 ± 0.9	3.2 ± 0.9	3.2 ± 0.9	3.2 ± 0.8	0.879
eGFR, mL/min/1.73 m^2^	45.1 ± 34.0	43.8 ± 33.9	54.7 ± 36.4	36.8 ± 29.3	0.003^§^
UACR, g/g	4.0 ± 5.4	4.4 ± 6.5	4.7 ± 6.8	3.4 ± 3.7	0.336
UPCR, g/g	5.3 ± 6.7	6.1 ± 6.7	5.2 ± 7.7	4.8 ± 5.5	0.480

† *Post-hoc test of LDL-C showed only a significant difference between middle tertile and highest tertile (P = 0.029)*.

§ *Post-hoc test of eGFR showed only a significant difference between middle tertile and highest tertile (P = 0.002)*.

*Gal-3, galectin-3; LDL-C, low density lipoprotein-cholesterol eGFR, estimated glomerular filtration rate; UACR, urine albumin-creatinine ratio; UPCR, urine protein-creatinine ratio*.

### The Associations Between Plasma Gal-3, Survival and CKD

In our study, non-survivors were found to have significantly higher levels of plasma Gal-3 than survivors (1678.9 ± 725.2 pg/mL vs. 944.8 ± 563.9 pg/mL; *P* = 0.005; Supplementary Figure 1A). Supplementary Table 1 shows the demographic and clinical characteristics of our patients stratified by the presence and absence of CKD. Among the study participants, 180 (72.3%) were in the CKD group (defined as eGFR <60 ml/min/1.73 m^2^), and 69 (27.7%) were in the non-CKD group. Patients in the CKD group had significantly higher levels of plasma Gal-3 than those in the non-CKD group (1016.3 ± 628.1 pg/mL vs. 811.6 ± 369.6 pg/mL; *P* = 0.010; Supplementary Figure 1B). In multivariate logistic regression analysis, increment of plasma Gal-3 level was significantly associated with the risks of CKD (per 100 pg/mL increases: adjusted odds ratio 1.07; 95% confidence interval 1.01–1.14; *P* = 0.032; Supplementary Table 2).

### Correlations of Gal-3 With EGFR, Creatinine, UPCR, and UACR

**Figure 1:**
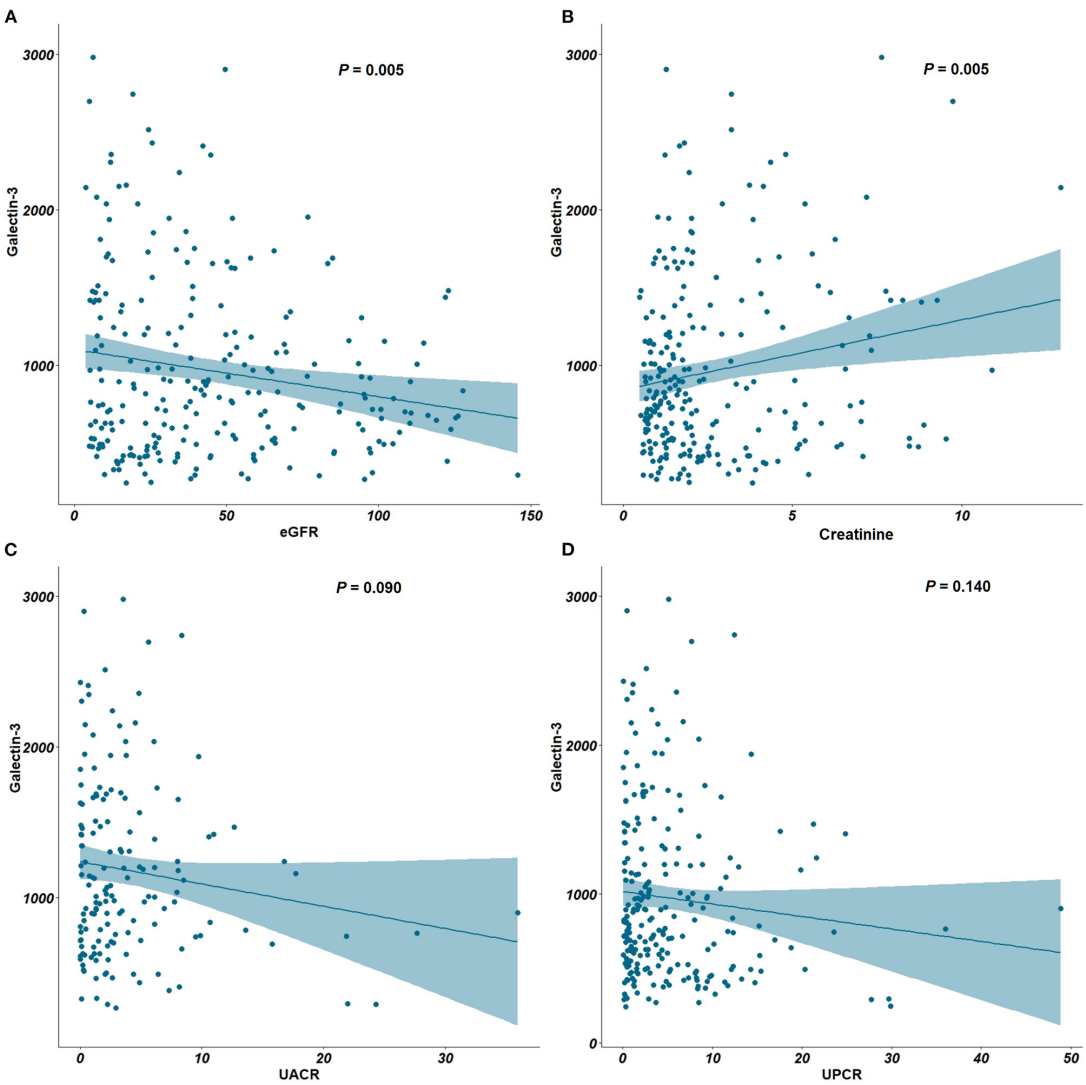
Correlation between plasma Gal-3, eGFR, creatinine, UACR and UPCR. **(A)** Correlation plots between Gal-3 and eGFR. **(B)** Correlation plots between Gal-3 and creatinine. **(C)** Correlation plots between Gal-3 and UACR. **(D)** Correlation plots between Gal-3 and UPCR. Gal-3, Galectin-3; eGFR, estimated glomerular filtration rate; UACR, urine albumin-creatinine ratio; UPCR, urine protein-creatinine ratio.

An inverse correlation between Ga1-3 levels and eGFR (*P* = 0.005) and a positive correlation between Gal-3 levels and serum creatinine (*P* = 0.005) were found (Figures 1A,B). Notably, while plasma Gal-3 levels appeared to have an inverse correlation with eGFR, the UACR and UPCR were not significantly correlated with plasma Gal-3 levels (Figures 1C,D).

### Plasma Gal-3 and Annual Change of EGFR in Non-CKD Patients

**Figure 2:**
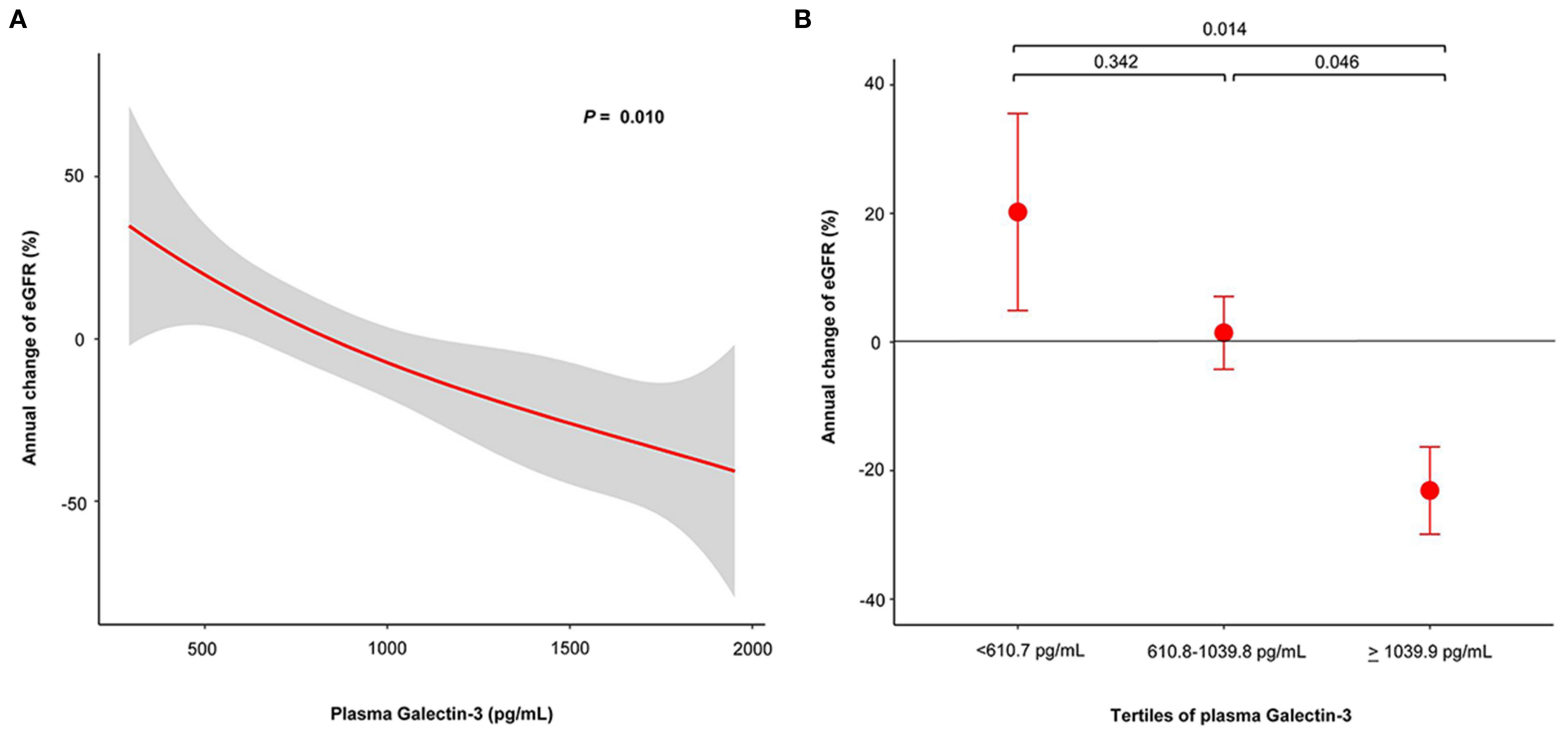
Plasma Gal-3 predicts risk of annual change of eGFR in non-CKD patients whose eGFR > 60 ml/min/1.73 m^2^. **(A)** Restricted cubic spline plots of the association between annual change of eGFR and plasma Gal-3 levels. The red line indicates the change of eGFR with the respective 95% confidence interval (gray area). **(B)** Annual change of eGFR according to tertile of plasma Gal-3 levels. Gal-3, Galectin-3; eGFR, estimated glomerular filtration rate; CKD, chronic kidney disease.

In non-CKD patients, we analyzed the associations between plasma Gal-3 and eGFR change. Figure 2A shows the restricted cubic spline plots displaying the association between plasma Gal-3 levels and the annual change of eGFR. The higher levels of plasma Gal-3 were associated with significantly greater loss of eGFR. Based on the tertile of plasma Gal-3 levels, the annual change of eGFR showed increase in the lowest tertile of plasma Gal-3, stable in the middle tertile of plasma Gal-3 and decline in the highest tertile of plasma Gal-3. Highest tertile of plasma Gal-3 was associated with significantly greater loss of eGFR compared to middle and lowest tertile of plasma Gal-3 levels (Figure 2B).

### Histopathologic Analysis of Kidney Biopsies

**Figure 3:**
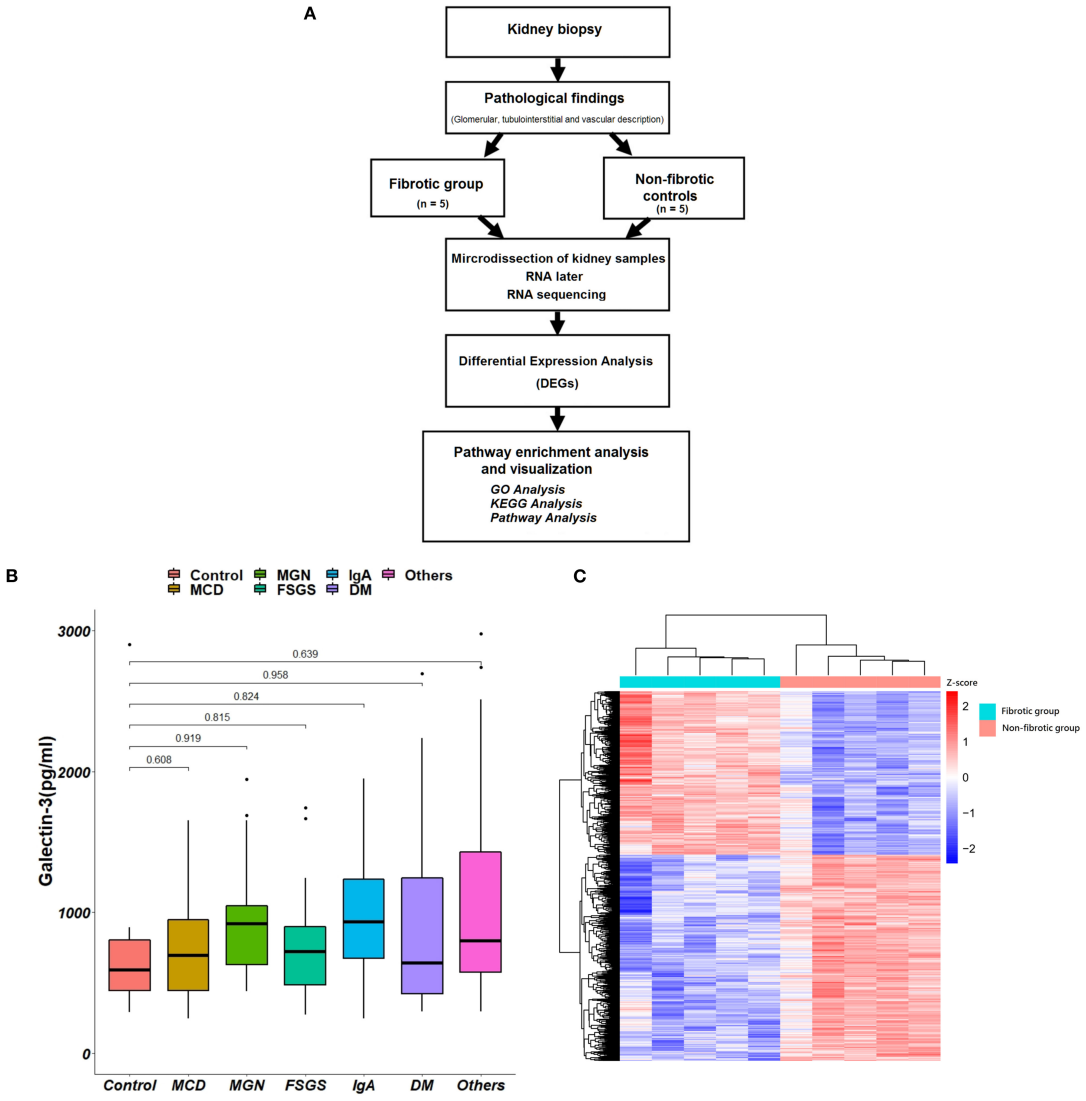
Kidney biopsy specimens for pathology and RNA sequencing analysis. **(A)** The algorithm of the collections of kidney biopsy specimens. **(B)** The associations between different pathological diagnoses and plasma Gal-3 levels. **(C)** The hierarchical clustering heat map shows the expression of different genes between fibrotic groups and non-fibrotic controls. Gal-3, galectin-3; GO, Gene Ontology; KEGG, Kyoto Encyclopedia of Genes and Genomes; MCD, minimal change disease; FSGS, focal segmental glomerulosclerosis; IgA, immunoglobulin A; DM, diabetes mellitus.

The algorithm of collected kidney biopsy specimens sent for pathology and RNA sequencing analysis is shown in Figure 3A. Plasma Gal-3 levels were not significantly different among the different pathological diagnoses (Figure 3B). The associations between different pathological diagnoses and eGFR, UACR and UPCR are shown in Supplementary Figure 2.

As shown in Table 2, we found that higher Gal-3 levels were significantly associated with GBM double contour (*P* < 0.001), GBM rigid (*P* = 0.029), glomerular necrosis (*P* = 0.017), and extracapillary hypertrophy (*P* = 0.039). Higher Gal-3 levels were associated with interstitial fibrosis (*P* = 0.028) and tubular atrophy (*P* = 0.032). For vascular structure, higher Gal-3 levels were also associated with increased vascular intimal fibrosis (*P* < 0.001) and vascular necrosis (*P* = 0.017).

**Table 2:** Regression for plasma Gal-3 and renal pathological findings.

**Pathological findings**	**Coefficient**	**Confidence interval**	***R* ^**2**^**	***P*-value**
**Glomerular description**				
Global sclerosis	−24.053	(−209.491–161.386)	<0.001	0.798
Segmental sclerosis	58.98	(−127.64–245.599)	0.002	0.534
GBM double contour^*^	**374.419**	**(169.659**–**579.179)**	**0.072**	**<0.001**
GBM rigid^*^	**196.816**	**(20.307**–**373.324)**	**0.028**	**0.029**
GBM thickening	146.028	(−32.871–324.927)	0.015	0.109
GBM collapse	431.721	(−154.56–1018.002)	0.012	0.148
GBM attenuation	−627.665	(−1451.513–196.183)	0.013	0.134
Glomerular necrosis^*^	**1400.391**	**(250.475**–**2550.306)**	**0.033**	**0.017**
Glomerular inflammatory change	−22.687	(−221.093–175.719)	<0.001	0.822
Glomerular ischemic change	94.514	(−84.13–273.157)	0.006	0.298
Endocapillary hypertrophy	159.06	(−18.749–336.869)	0.018	0.079
Extracapillary hypertrophy^*^	**187.167**	**(9.459**–**364.875)**	**0.025**	**0.039**
Mesangial hypertrophy	129.668	(−64.649–323.985)	0.011	0.189
Mesangial matrix expansion	179.371	(−18.488–377.23)	0.019	0.075
Mesangial matrix mesangiolysis	188.558	(−30.578–407.694)	0.017	0.091
**Tubulointerstital descriptions**				
Interstitial inflammation	1.526	(−3.388 – 6.441)	0.002	0.541
Tubulitis	−22.687	(−221.093–175.719)	<0.001	0.822
Interstitial edema	−505.854	(−1180.664–168.957)	0.013	0.141
Interstitial fibrosis^*^	**4.714**	**(0.505 – 8.922)**	**0.028**	**0.028**
Tubular atrophy^*^	**4.61**	**(0.398 – 8.823)**	**0.027**	**0.032**
Acute tubular necrosis	3.068	(−177.883–184.02)	<0.001	0.973
Casts	−6.142	(−1175.555–1163.271)	<0.001	0.992
Calcinosis	−305.517	(−893.625–282.592)	0.006	0.307
**Vasculature descriptions**				
Hyaline arteriosclerosis	34.72	(−144.645–214.084)	0.001	0.703
Vascular intimal fibrosis^*^	**398.614**	**(206.866–590.362)**	**0.091**	**<0.001**
Vascular medial proliferation	−273.066	(−1441.744–895.613)	0.001	0.645
Vascular necrosis^*^	**1400.391**	**(250.475–2550.306)**	**0.033**	**0.017**

* *Statistically significant and P-value was shown in bold*.

*Gal-3, galectin-3; GBM, glomerular basement membrane*.

### RNA-Sequencing Analysis Identifies the Gene Expression of Gal-3

We compared the transcriptome profile of renal tubulointerstitial compartments between fibrotic kidney biopsy samples (*n* = 5; the degree of interstitial fibrosis > 50%) and non-fibrotic controls (*n* = 5). Heatmaps of the up- and downregulated genes among the DEGs are presented in Figure 3C.

### Gene Ontology Analysis

**Figure 4:**
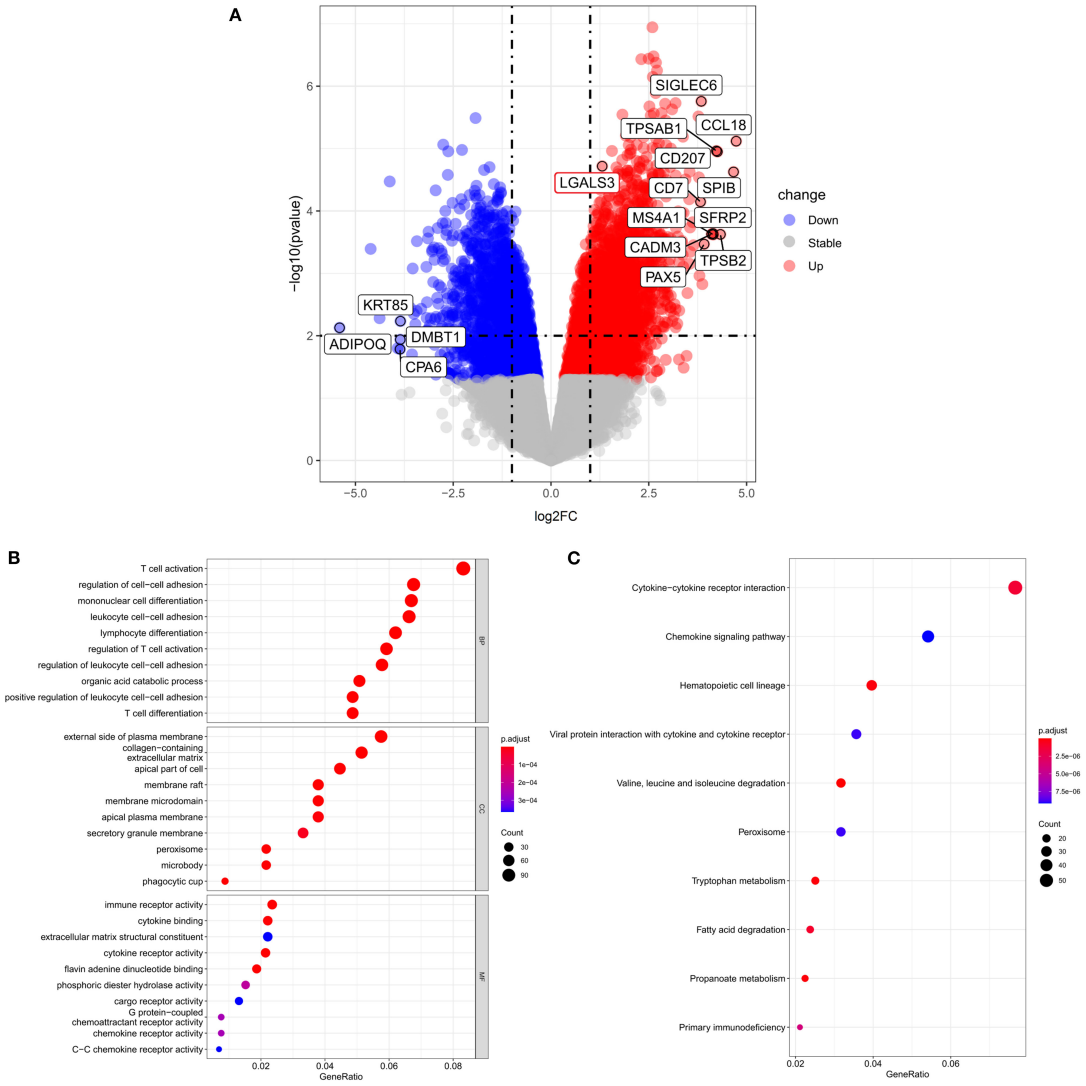
Volcano plots and GO and KEGG enrichment analysis according to RNA sequence analysis. **(A)** Volcano plot of differentially expressed genes between fibrotic groups and non-fibrotic controls. **(B)** The biological process, cellular components and molecular function identified from the GO and **(C)** KEGG enrichment analysis of the RNA sequencing. GO, Gene Ontology; KEGG, Kyoto Encyclopedia of Genes and Genomes.

A total of 5,644 DEGs were identified, including 3,164 upregulated genes and 2,480 downregulated genes in fibrotic samples compared to non-fibrotic controls. The volcano plot of the DEGs is shown in Figure 4A. The Gal-3 gene (LGALS3) was upregulated in fibrotic groups compared to non-fibrotic groups. We further performed GO enrichment analysis (Figure 4B). Regarding the BPs, the DEGs were mainly enhanced for T cell activation, regulation of cell-cell adhesion, monocyte cell differentiation and leukocyte cell-cell adhesion involved in the immune response. For the CC functional group, DEGs were enriched on the external side of plasma membrane, collagen-containing extracellular matrix and apical part of the cells, etc. Moreover, for the MF functional group, DEGs were enhanced in immune receptor activity and cytokine binding, etc.

### KEGG Analyses and Pathway Enrichment Analysis

KEGG analyses (Figure 4C) indicated that the top three KEGG pathways included cytokine-cytokine receptor interactions, chemokine signaling pathways, and hematopoietic cell lineages. The KEGG pathways were further visualized by enrichmap according to overlapping gene sets. The Gal-3-encoded gene LGALS3 was connected by both the leukocyte cell-cell adhesion pathway and the regulation of T cell activation pathways (Supplementary Figure 3).

## Discussion

Our study aims to explore the association between the expression of Gal-3, pathological findings of kidney biopsy and RNA-sequencing analysis. First, we found that higher Gal-3 levels were associated with an increased risk of renal fibrosis, and Gal-3 was inversely associated with eGFR. Second, we further demonstrated that Gal-3 was associated with interstitial fibrosis, tubular atrophy and vascular intimal fibrosis. Finally, the expression of Gal-3 was upregulated in fibrotic kidney biopsy samples in RNA-sequence analysis. GO enrichment analysis found that the DEGs were mainly enhanced in T cell activation, monocyte cell differentiation, and the regulation of cell-cell adhesion. KEGG analyses also found an association between cytokine-cytokine receptor interactions and chemokine signaling pathways. Taken together, our data suggest that Gal-3 may be associated with a risk of lower eGFR and the development of renal fibrosis through the involved immune response and pathway.

The Framingham Offspring Study, which investigated 2,450 participants from the general population with 10 years of follow-up, found that higher levels of Gal-3 were associated with CKD despite adjustment for other confounding factors (21). The Atherosclerosis Risk in Communities Study consisted of 9,148 African American and white men and women without pre-existing heart failure. Higher plasma Gal-3 levels were also associated with an increased risk of incident CKD, and the association became stronger in patients with hypertension (35). In contrast, however, in the Cardiovascular Heart Study including 2,763 subjects, the association between baseline Gal-3 levels and CKD was not statistically significant in the final analyses (36). Our study provided support that Gal-3 was inversely associated with eGFR in our study participants.

A hospital-based study including 205 outpatients found that Gal-3 levels were considered an early marker for renal dysfunction and the progression of cardiorenal syndrome in heart failure. Stepwise regression analysis found that Gal-3 was not associated with microalbuminuria (37). In contrast, the Framingham Offspring Study including 2,450 participants from 1995 to 2008 found that higher Gal-3 levels were associated with higher risks of incident CKD but were not associated with increased risks of albuminuria. Similar to the findings of the Framingham Offspring Study, our study found that Gal-3 was not associated with either the UPCR or UACR.

Although Gal-3 might play a causal role in tissue remodeling and the development of organ fibrosis (38), a previous study focused on kidney biopsy findings in humans remains scarce (39). In our study, we found that Gal-3 was associated with interstitial fibrosis, tubular atrophy and vascular intimal fibrosis, irrespective of the etiology of renal diseases. Gal-3 is widely expressed in immune cells (40–42), especially in monocytes/macrophages, which profoundly affect critical macrophage functions, such as phagocytosis and phenotype transition (43–45). A mouse model of unilateral ureteral obstruction showed that Gal-3 expression was upregulated, and depletion of Gal-3 protected against the accumulation of renal myofibroblasts and further fibrosis (45). Profibrotic microphages also demonstrated upregulation of several profibrotic genes to stimulate the production of fibronectin and extracellular matrix proteins (46). In addition, extracellular Gal-3 also has the ability to regulate T cell function to regulate further immune responses during fibrotic processes (47, 48). In our RNA sequence analysis, we found that LGALS3 was significantly upregulated in fibrotic groups compared to controls. For the GO enrichment analyses, the DEGs were mainly enhanced for monocyte cell differentiation, T cell activation and cell-cell adhesion. The KEGG pathways also showed that DEGs were enhanced in cytokine-cytokine receptor interactions and chemokine signaling pathways. Laminin, fibronectin and integrins are known ligands for Gal-3, and therefore, Gal-3 may promote pathological fibrotic processes by regulating cell-cell adhesion and cell proliferation (49, 50). Therefore, our study may provide some evidence that Gal-3 plays a multifaceted role in the regulation of inflammatory responses to promote cell-cell interactions and adhesions, which contributes to renal fibrosis that is associated with renal failure.

The strengths of our study are the well-characterized sample, completeness of the data collection and verification of participating patients. Our study showed a positive correlation between Gal-3 and the risk of renal fibrosis. We provided new data indicating that patients with higher levels of Gal-3 have a significantly higher risk of interstitial fibrosis, vascular intimal fibrosis and tubular atrophy than those with lower levels of Gal-3. Another major strength of this study is that it provides RNA-sequencing evidence of the gene expression of Gal-3 and renal pathological findings, which lends further credence to the study findings.

Our study has some limitations that should be considered. All eGFRs were measured once at the time of kidney biopsy. Therefore, it is possible that acute kidney injury or variability in renal function may not have been detected in our analyses. However, all participants in our study had undergone kidney biopsy, and therefore, the definitive etiology of CKD may be assured. Detailed biopsy information, such as the degree of tubular atrophy and interstitial fibrosis, was also identified in our study. Second, the data of our participants were based on a tertiary medical center, which may include more complicated cases with more underlying diseases, and thus, this may limit the generalizability of our results. Finally, we did not perform serial measurements of plasma Gal-3 over time, so whether the change in Gal-3 was associated with renal function progression was unknown. Further prospective studies may be needed to confirm our results and the potential ability of Gal-3 as a biomarker of CKD progression.

In conclusion, we found an association between plasma Gal-3 and the risk of renal fibrosis in kidney biopsy specimens. Gal-3 may be considered a potential biomarker for detecting renal fibrosis, potentially affording an opportunity for early intervention to prevent disease progression. However, the precise mechanisms of Gal-3 in the development of CKD warrant further investigation.

## Data Availability Statement

The datasets presented in this study can be found in online repositories. The names of the repository/repositories and accession number(s) can be found below: https://www.ncbi.nlm.nih.gov/bioproject/770300. Accession number(s):PRJNA770300.

## Ethics Statement

The studies involving human participants were reviewed and approved by Taipei Veteran General Hospitals (2018-06-008B). The patients/participants provided their written informed consent to participate in this study.

## Author Contributions

S-MO, M-TT, H-YC, F-AL, W-CT, K-HL, F-PC, Y-PL, R-BY, and D-CT: conception and study design. S-MO, M-TT, H-YC, F-AL, W-CT, K-HL, F-PC, and D-CT: data acquisition. S-MO, M-TT, R-BY, and D-CT: data analysis/interpretation and statistical analysis. S-MO, M-TT, H-YC, F-AL, W-CT, K-HL, F-PC, Y-PL, R-BY, and D-CT: drafting of the manuscript. All authors contributed to the article and approved the submitted version.

## Funding

This work was supported in part by the Ministry of Science and Technology, Taiwan (MOST 106-2314-B-010-039-MY3, MOST 107-2314-B-075-052, MOST 108-2314-B-075-008, MOST 109-2314-B-075−067-MY3, MOST 109-2320-B-075-006, MOST 110-2321-B-A49-003); Taipei Veterans General Hospital (V107B-027, V108B-023, V108C-103, V108D42-004-MY3-1, V108D42-004-MY3-2, V108D42-004-MY3-3, V109B-022, V109C-114, V109D50-001-MY3-1, V109D50-001-MY3-2, V110C-152, V110E-003-2); Taipei, Taichung, Kaohsiung Veterans General Hospital, Tri-Service General Hospital, Academia Sinica Joint Research Program (VTA110-V1-3-1); Taipei Veterans General Hospital-National Yang-Ming University Excellent Physician Scientists Cultivation Program (No. 104-V-B-044); Institute of Biomedical Sciences, Academia Sinica (IBMS-CRC110-P04 and AS-VTA-110-03) and Foundation for Poison Control (FPC-109-002).

## Conflict of Interest

The authors declare that the research was conducted in the absence of any commercial or financial relationships that could be construed as a potential conflict of interest.

## Publisher's Note

All claims expressed in this article are solely those of the authors and do not necessarily represent those of their affiliated organizations, or those of the publisher, the editors and the reviewers. Any product that may be evaluated in this article, or claim that may be made by its manufacturer, is not guaranteed or endorsed by the publisher.
