# Supplementary material for: Identification of Galectin-3 as Potential Biomarkers for Renal Fibrosis by RNA-Sequencing and Clinicopathologic Findings of Kidney Biopsy
Source: Front Med (Lausanne). 2021 Nov 12;8:748225. doi: 10.3389/fmed.2021.748225 (PMC8633540; doi:10.3389/fmed.2021.748225)
Supplement: Supplementary file 1 [file Data_Sheet_1.docx]

**Identification of Galectin-3 as potential biomarkers for renal fibrosis by RNA-sequencing and clinicopathologic findings of kidney biopsy**

**Authors:** Shuo-Ming Ou, Ming-Tsun Tsai, Huan-Yuan Chen, Fu-An Li, Wei-Cheng Tseng, Kuo-Hua Lee, Fu-Pang Chan, Yao-Ping Lin, Ruey-Bing Yang and Der-Cherng Tarng

**Contents**

| **Supplemental Figures** |  |
| --- | --- |
| Supplementary Table 1. Clinical characteristics of our study participants stratified by CKD and non-CKD groups | 2 |
| Supplementary Table 2. Multivariate logistic regression analysis for risks of CKD | 3 |
| **Supplemental Figures** |  |
| Supplementary Figure 1. The associations between plasma Gal-3, survival and CKD | 4 |
| Supplementary Figure 2. The associations between different pathological diagnoses and (A) eGFR, (B) UACR and (C) UPCR | 5 |
| Supplementary Figure 3. Coexpression networks of Gal-3-encoded gene LGALS3 and coregulated pathways. | 6 |

| **Supplementary Table 1. Clinical characteristics of our study participants stratified by CKD and non-CKD groups** | | | | |
| --- | --- | --- | --- | --- |
|  | **All patients** | **CKD groups†** | **Non-CKD groups** | ***P* value** |
|  | **(*n* = 249)** | **(*n* =** **180)** | **(*n* = 69)** |  |
| **Age, years** | 57.2 ± 16.3 | 59.7 ± 15.7 | 50.6 ± 16.2 | <0.001 |
| **Male sex, *n*(%)** | 155 (62.3) | 117 (65) | 38 (55.1) | 0.148 |
| **Body mass index** | 25.6 ± 4.6 | 25.4 ± 4.4 | 26.1 ± 5.1 | 0.263 |
| **Systolic blood pressure, mmHg** | 136.8 ± 22.7 | 140 ± 23.1 | 128.4 ± 19.4 | <0.001 |
| **Diastolic blood pressure, mmHg** | 77.2 ± 13.0 | 77.7 ± 13.5 | 75.7 ± 11.9 | 0.263 |
| **Diabetes mellitus, *n*(%)** | 64 (25.7) | 53 (29.4) | 11 (15.9) | 0.029 |
| **Coronary artery disease, *n*(%)** | 20 (8.0) | 17 (9.4) | 3 (4.4) | 0.185 |
| **Congestive heart failure, *n*(%)** | 46 (18.5) | 39 (21.7) | 7 (10.1) | 0.036 |
| **Hypertension, *n*(%)** | 105 (42.2) | 90 (50) | 15 (21.7) | <0.001 |
| **Dyslipidemia, *n*(%)** | 47 (18.9) | 36 (20) | 11 (15.9) | 0.464 |
| **Malignancy, *n*(%)** | 60 (24.1) | 54 (30) | 6 (8.7) | <0.001 |
| **LDL-C, mg/dL** | 140.5 ± 85.6 | 117.8 ± 61.7 | 198.2 ± 108.6 | <0.001 |
| **Uric acid, mg/dL** | 6.7 ± 2.2 | 6.8 ± 2.3 | 6.6 ± 1.8 | 0.603 |
| **Albumin, mg/dL** | 3.2 ± 0.9 | 3.3 ± 0.8 | 2.9 ± 0.9 | 0.001 |
| **eGFR, mL/min/1.73 m^2^** | 45.1 ± 34.0 | 26.9 ± 16.3 | 91.4 ± 20.8 | <0.001 |
| **UACR, g/g** | 4.0 ± 5.4 | 3.9 ± 5.4 | 4.3 ± 5.7 | 0.714 |
| **UPCR, g/g** | 5.3 ± 6.7 | 5.5 ± 6.9 | 4.9 ± 6.1 | 0.575 |
| †CKD group was defined as patients whose eGFR <60 mL/min/1.73m^2^.  *Abbreviation*s: CKD, chronic kidney disease; LDL-C, low density lipoprotein-cholesterol; eGFR, estimated glomerular filtration rate; UACR, urine albumin-creatinine ratio; UPCR, urine protein-creatinine ratio. | | | | |

| **Supplementary Table 2. Multivariate logistic regression analysis for risks of CKD** | | | |
| --- | --- | --- | --- |
|  | **Increment of plasma Gal-3 (per 100 pg/mL)** | | |
|  | **Odds Ratio** | **95% CI** | ***P* value** |
| **Model 1** | 1.074 | 1.02-1.14 | 0.014 |
| **Model 2** | 1.071 | 1.01-1.14 | 0.032 |
| Model 1 was unadjusted  Model 2 was adjusted for age, diabetes mellitus, heart failure, hypertension, Malignancy, LDL-C and albumin.  *Abbreviation*: CKD, chronic kidney disease; Gal-3, galectin-3; CI, confidence interval; LDL-C, low density lipoprotein-cholesterol. | | | |

**Supplementary Figure 1. The associations between plasma Gal-3, survival and CKD. (A) Plasma Gal-3 levels in survivor versus non-survivor groups (1678.9 ± 725.2 pg/mL vs. 944.8 ± 563.9 pg/mL; *P* = 0.005) (B) Plasma Gal-3 levels in CKD versus non-CKD groups (1016.3 ± 628.1 pg/mL vs. 811.6 ± 369.6 pg/ml; *P* = 0.010).**

*Abbreviation.* Gal-3, galectin-3; CKD, chronic kidney disease.

**
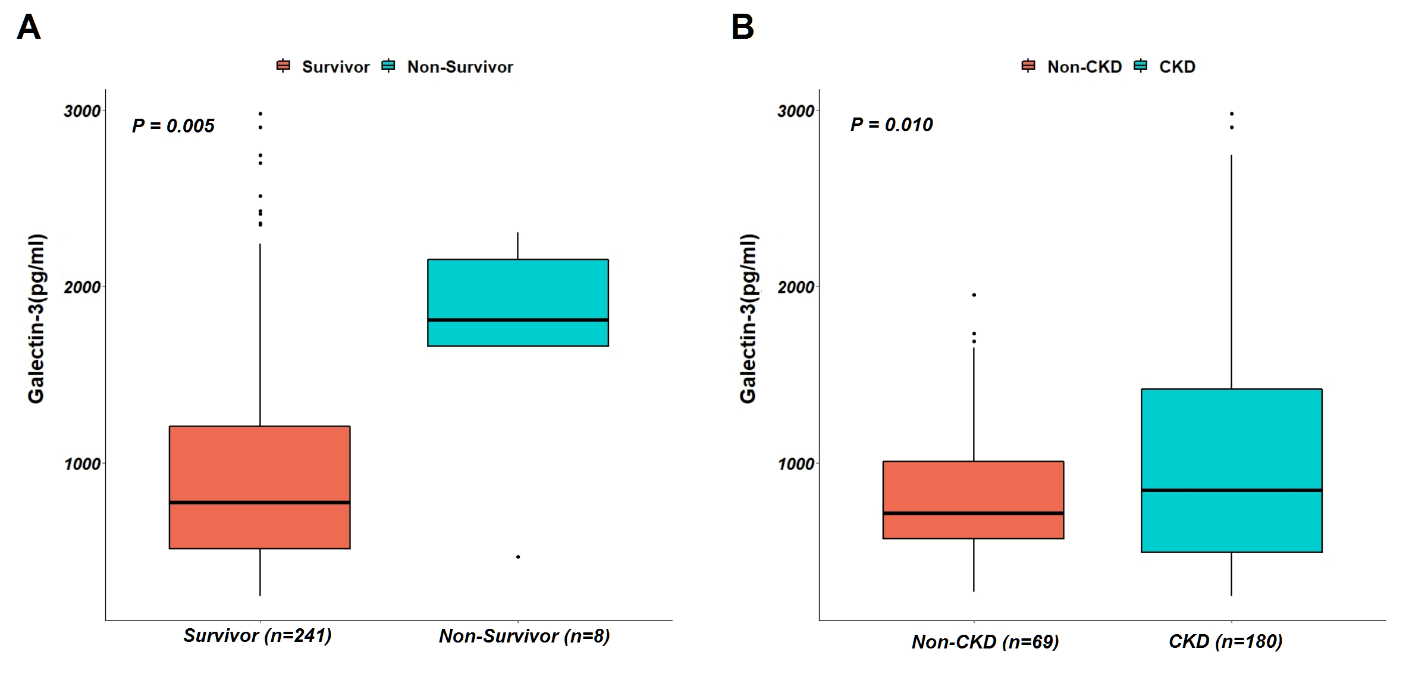
**

**Supplementary Figure 2. The associations between different pathological diagnoses and (A) eGFR, (B) UACR and (C) UPCR**

*Abbreviation.* eGFR estimated glomerular filtration rate; MCD, minimal change disease; FSGS, focal segmental glomerulosclerosis; IgA, immunoglobulin A; DM, diabetes mellitus; UACR, urine albumin-creatinine ratio; UPCR, urine protein-creatinine ratio.

**
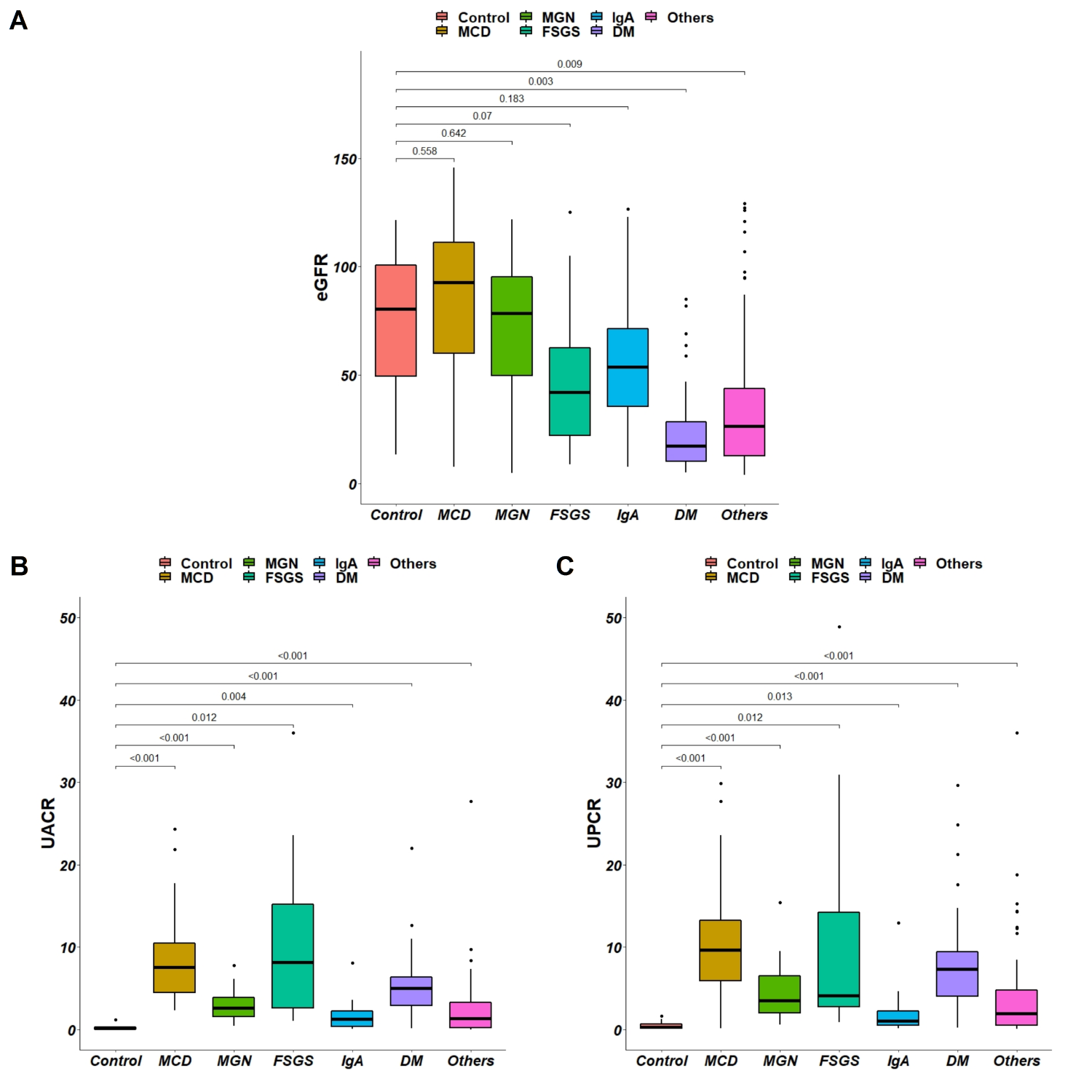
**

**Supplementary Figure 3. Coexpression networks of Gal-3-encoded gene LGALS3 and coregulated pathways.**

**
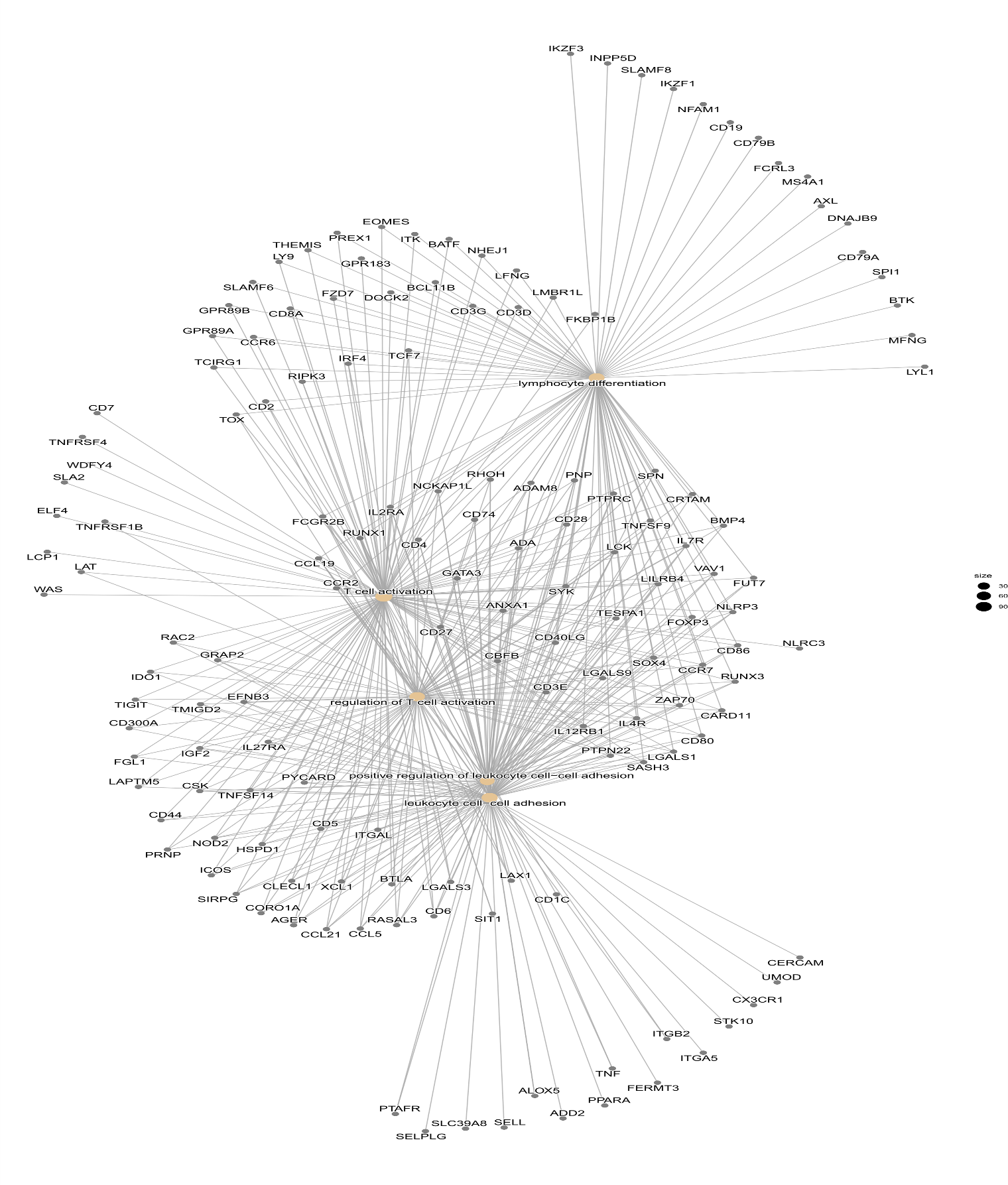
**
